# Structural Changes and Evolution of Peptides During Chill Storage of Pork

**DOI:** 10.3389/fnut.2020.00151

**Published:** 2020-09-22

**Authors:** Xiaoyu Zou, Jing He, Di Zhao, Min Zhang, Yunting Xie, Chen Dai, Chong Wang, Chunbao Li

**Affiliations:** ^1^Key Laboratory of Meat Processing and Quality Control, Ministry of Education, Nanjing Agricultural University, Nanjing, China; ^2^Jiangsu Collaborative Innovation Center of Meat Production and Processing, Quality and Safety Control, Nanjing Agricultural University, Nanjing, China; ^3^Key Laboratory of Meat Processing, Ministry of Agriculture and Rural Affairs, Nanjing Agricultural University, Nanjing, China; ^4^Experimental Teaching Center of Life Science, Nanjing Agricultural University, Nanjing, China; ^5^National Center for International Research on Animal Gut Nutrition, Nanjing Agricultural University, Nanjing, China; ^6^Joint International Research Laboratory of Animal Health and Food Safety, Ministry of Education, Nanjing Agricultural University, Nanjing, China

**Keywords:** pork, chill storage, *in vitro* digestion, Raman spectroscopy, LC-MS/MS

## Abstract

In this work, we investigated changes in protein structures in vacuum-packed pork during chill storage and its impact on the *in vitro* protein digestion. *Longissimus dorsi* muscles were vacuum packed and stored at 4°C for 3 days. Samples were subjected to Raman spectroscopy, *in vitro* digestion and nano LC-MS/MS. The 3 d samples had lower α-helix content, but higher β-sheet, β-turn, and random coil contents than the 0 d samples (*P* < 0.05). SDS-PAGE revealed significant protein degradation in the 3 d samples and the differences in digested products across the storage time. Proteome analysis indicated that the 3 d samples had the higher susceptibility to digestion. Increasing protein digestibility was mainly attributed to the degradation of myofibrillar proteins. Thus, exposure of more enzymatic sites in loose protein structure during chill storage could increase protein degradation in meat.

## Introduction

Postmortem aging and its impact on eating quality of fresh meat has been widely concerned (1–3). Physicochemical and structural changes in meat proteins occurring in postmortem aging and processing may affect the protein digestion and nutritional value of meat (4–6). Postmortem aging for relatively long time may produce bioactive peptides in beef that exhibit DPPH radical scavenging, ACE- and renin-inhibitory activities (7). However, little is known about whether bioactive peptides are produced in a short-term aging of pork.

At early postmortem time, the dephosphorylation of several metabolic enzymes has been shown to affect the rate of glycolysis and pH decline, and subsequently the activation of μ-calpain, the release of lysosomal enzymes and meat tenderization (8). In addition, protein oxidation may occur in fresh meat during chill storage (9), which may cause the formation of disulfide bonds, and a decrease in protein hydrophilicity and water holding capacity. To avoid such changes, vacuum packaging and chill storage have been widely applied (10). However, protein oxidation and structural changes may still occur in vacuum-packed and chilled meat because radical oxygen species widely existing in living muscle tissues could be retained in postmortem muscles (11). Furthermore, protein oxidation also alters the protein structures and digestibility of meat (5). An increase in digestion can be attributed to protein unfolding and increased susceptibility to digestion (4). Previous studies have focused how processing and physicochemical changes affect protein structure and digestion (12, 13). However, few data are available on how structural changes of meat proteins enhanced digestibility of fresh meat from a proteomic perspective, in terms of the evolution of peptides from the stomach to the small intestine. In this study, we investigated the structural changes of meat proteins in pork during chill storage and their impacts on protein digestibility and the release of bioactive peptides.

## Materials and Methods

### Reagents

Ellman's reagent (4 mg/mL 5,5′-dithiobis-2-nitrobenzoic acid in Tris-Gly) and Tris-Gly (10.4 g Tris, 6.9 g glycine, and 1.2 g EDTA per liter, pH 8.0) were obtained from Sigma Aldrich (St. Louis, MO, USA), as were porcine gastric pepsin (Cat. No. P7125) and porcine pancreatic trypsin (Cat. No. T7409). BCA protein assay kit (No. 23225) and protein marker (No. 26619) were obtained from Thermo Scientific (Rockford, IL, USA). Amicon Ultracel-3 membrane (UFC500396) and Zip Tip C18 pipette tips (ZTC18S096) were obtained from Millipore (Billerica, MA, USA).

### Sample Preparation

*Longissimus dorsi* muscles (size: 10 × 10 × 5 cm) were obtained from 8 native Suhuai pig carcasses at the same line in a commercial slaughterhouse. Each muscle was cut into four 2.5 × 10 × 5 cm pieces (weight: 50 to 65 g each), vacuum-packed and stored at 4°C for 3 days. Samples were taken on 0, 1, 2, and 3 d for further analyses.

### Raman Spectroscopy

Raman spectroscopy was performed to evaluate changes in protein secondary structure (LabRAM HR Evolution, Horiba/Jobin, Yvon, Longjumeau, France) as previously described (5). Laser (excitation wavelength: 785 nm, power: 100 mW) was applied and the backscattering Raman signals ranging from 400 to 3,200 cm^−1^ were collected. Raman spectra were normalized against the band at 1,003 cm^−1^. The 1,685–1,645 and 1,309–1,229 cm^−1^ bands correspond to amide I and III vibrational modes of α-helix, random coil, and β-sheet. The 1,341 and 940 cm^−1^ bands reflect CH-bending and C-C stretching respectively. The range of 1,658–1,650 cm^−1^ in the amide I vibrational mode mainly reflects C=O stretching vibrations, C-N stretching, and N-H in-plane bending of peptide groups. The 1,003, 830–850, and 760 cm^−1^ bands correspond to phenylalanine, tyronsine, and tryptophan, respectively. The α-helix, β-sheet, β-turn, and random coil were quantified (14).

### *In vitro* Digestion

The protein digestibility of cooked meat from 0, 1, 2 to 3 d samples was assessed as described by Zou et al. (15). Briefly, meat pieces were packed in plastic bags and cooked in a 72°C water bath until the core temperature reached 70°C. The cooking time was around 20 min. The core temperature was tracked by a portable thermal probe (Pt 100, Testo AG, Mönchaltorf, Schweiz, Germany). After cooking, meat samples were cooled for 2 h to room temperature (22°C). Then, samples (1.00 g) were homogenized (9,600 rpm, 30 s, twice; 13,400 rpm, 30 s, twice) in PBS (10 mmol/L, pH 7.0). The homogenate was digested by gastric pepsin (32 mg/mL in 0.1 mol/L HCl, pH 1.0) at 37°C for 2 h and the digestion was stopped by adjusting the pH to 7.5 with 1 mol/L NaOH. Then the resulting mixture was digested by trypsin (24 mg/mL in 0.1 mol/L PBS, pH 7.0) at 37°C for 2 h and stopped by heating the system at 95°C for 5 min.

The undigested proteins were precipitated by adding three volumes of ethanol at 4°C for 12 h. Then the samples were centrifuged at 10,000 × g at 4°C for 20 min. Protein contents in the whole meat samples and the precipitates were quantified by a BCA protein assay kit (Thermo Scientific, Rockford, IL) according to the manufacturer's instructions. Protein digestibility was calculated as follows:

$$\begin{array}{l} \text{Protein digestibility }\left(\%\right)=\frac{W0-W1}{W0}\times 100\% \end{array}$$

Where *W*_1_ is the content (g) of undigested proteins precipitated by ethanol. *W*_0_ is the total protein content (g) in the whole meat before digestion.

The supernatant containing digested products that are ethanol soluble was subjected to nano LC-MS/MS analysis and the precipitate including undigested proteins was separated on the SDS-PAGE gels.

### SDS-PAGE

The whole proteins were extracted according to the method of Zarkadas and Maloney (16) with some modifications. Briefly, cooked meat samples (0.3 g) were homogenized in 4.5 mL 2% SDS with 3 × 30 s at 10,000 rpm and centrifuged at 4,000 × g for 20 min at 4°C. Two milliliters of the supernatant were dialyzed for 48 h in 1 L distilled water to remove SDS. The dialyzed samples were transferred to new tubes and stored at −80°C for further analyses.

SDS-PAGE was performed to separate proteins or their fragments under reducing conditions (5). Appropriate volumes of samples were mixed with 12.5 μL sample buffer (4×) and 5 μL reducing sample agent (10×) and made up to a total volume of 50 μL with ultrapure water. The final protein concentrations were 0.50 μg/μL for all samples. The samples were heated at 70°C for 10 min. Twelve microliters of samples were loaded in triplicate into the wells of 4–12% precast gels (GenScript, Piscataway, NJ, USA). The gels were run in a total 800 mL of SDS running buffer at 150 V till the blue dye front disappeared. The gels were stained with colloidal Coomassie brilliant blue R250 (CBB) for 30 min and then destained for 20 min three times. CBB staining solution contained 0.1% CBB/45% acetic acid/10% ethanol/45% ultrapure water. Destaining buffer contained 10% acetic acid/10% ethanol/80% ultrapure water. The gel images were acquired by an image scanner (GE Healthcare, Little Chalfont, Uppsala, Sweden) and the band intensities were quantified with the Quantity One image analysis software (Bio-Rad, Hercules, CA). The relative intensities of bands were calculated by the actual band intensity divided by that of the 150 kDa band in the calibration marker lane.

### Protein Identification of Digested Products by Nano LC-MS/MS

The ethanol-soluble fractions of the digested products were identified by *Nano LC-MS/MS* system as previously described (17) with minor modifications. Briefly, ethanol in the supernatant was removed by a vacuum concentrator (ZXJY, Beijing, China), and dried in a freeze dryer (Christ, Osterode, Germany). The dried samples were dissolved in 0.2% formic acid in ultrapure water and then centrifuged (15,000 × g, 15 min, 4°C) in ultra-0.5 mL filter tubes and desalted in ZipTip C18 tips (Millipore, Billerica, MA). Peptides (1.5 μg) were separated in a C18 column (2 cm × 200 μm, 5 μm, Thermo Fisher Scientific, Palo Alto, CA) and then sequentially a C18 chromatographic column (75 μm × 100 mm, 3 μm, Thermo Fisher Scientific, Palo Alto, CA). Elution was applied by running a mixture of buffer A (0.2% formic acid in 60% acetonitrile) and buffer B (0.2% formic acid in ultrapure water) at 300 nL/min. The elution buffers were changed by 97%A from 0 to 10 min, 92%A from 10 to 70 min, 62%A from 70 to 72 min, 2%A from 72 to 82 min, and 97%A from 82 to 90 min. The eluted peptides were identified by a hybrid quadrupole orbitrap mass spectrometer equipped with a nanoelectrospray ionization source (Thermo Fisher Scientific, Palo Alto, CA). A full-scan mode was selected from 300 to 1,800 amu.

MS/MS spectra were matched using the Proteome Discoverer-1.4 (Thermo Fisher Scientific, Palo Alto, CA). Pepsin and pepsin/trypsin were selected in peptic and peptic/tryptic peptides database search, respectively. The parameters for searching were set as follows: MS/MS tolerance: 10 ppm; main search: 4.5 ppm; missed cleavage: 2; searching database: Sus scrofa under Uniprot (Uniprot-Sus scrofa.fasta, www.uniprot.org/); de-isotopic: TRUE; fixed modification: carbamidomethyl (cys); variable modification: oxidation (met), acetyl (protein N-term); label free quantification (LFQ): TRUE; decoy database pattern: reverse; LFQ min ratio count: 1; match between runs: 2 min; peptide false discovery rate (FDR): 0.01; and protein FDR: 0.01. Proteins that could not be annotated were not further used. BIOPEP database was used in the search of similar sequences previously identified showing ACE inhibitory and DPPIV inhibitory activity (http://www.uwm.edu.pl/biochemia/index.php/pl/biopep).

### Statistical Analysis

The effects of storage time on measured variables (α-helix, β-sheet, β-turn, random coil, ${\text{I}}_{760\text{cm}}^{-1}$/${\text{I}}_{1003\text{cm}}^{-1}$ and ${\text{I}}_{850\text{cm}}^{-1}$/${\text{I}}_{830\text{cm}}^{-1}$) were evaluated by Student's *t*-test using GraphPad Prism 7.0 (GraphPad Software, Inc., La Jolla). The effects of storage time on other measured variables were evaluated by analysis of variance (ANOVA) and the least significant means were compared by Tukey's *post-hoc t*-test. Data were presented as means and standard deviations. The means were considered significantly different if the *P-*value was smaller than 0.05. Venn diagrams (http://bioinformatics.psb.ugent.be/webtools/Venn/) were applied to analyze the differences of matched proteins among the four time points.

## Results and Discussion

### Changes in Raw Meat Protein Structures Detected by Raman Spectroscopy

**Figure 1:**
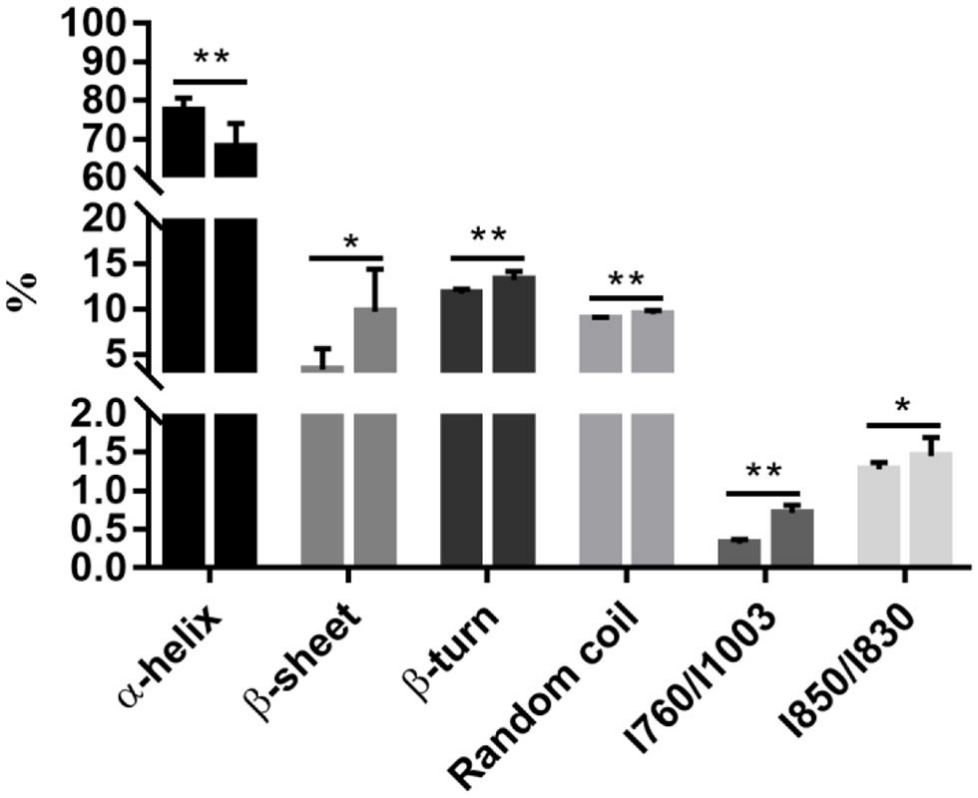
Postmortem aging caused a reduction of the α-helix content but an increase in the β-sheet, β-turn, and random coil contents, accompanying with the exposure of tryptophan. All data were presented as means and standard deviations. Asterisks denote that the contents of α-helix, β-sheet, β-turn, random coil, and the ratios of ${\text{I}}_{760\text{cm}}^{-1}$/${\text{I}}_{1003\text{cm}}^{-1}$ and ${\text{I}}_{850\text{cm}}^{-1}$/${\text{I}}_{830\text{cm}}^{-1}$ differ significantly between the two time points. *P < 0.05; **P < 0.05.

Raman spectroscopy provides powerful information on secondary structural modifications in proteins (18). The α-helix content decreased from 77.41% on 0 d to 67.89% on 3 d (*P* < 0.05, Figure 1), while β-sheet, β-turn, and random coil increased from 3.39, 11.72, and 8.92% on 0 d to 9.75, 13.21, and 9.49% on 3 d, respectively (*P* < 0.05, Figure 1). This indicates that denaturation and unfolding occurred in meat proteins from 0 to 3 d. During aging, meat proteins were degraded by endogenous enzymes into fragments. Degradation of myofibrillar proteins is modulated by protein oxidation and nitrosylation (19).

The side chains of tryptophan and tyrosine residues may change under a polar microenvironment, which results in modifications in the tertiary structure of proteins. The ratios of ${\text{I}}_{760\text{cm}}^{-1}$/${\text{I}}_{1003\text{cm}}^{-1}$ and ${\text{I}}_{850\text{cm}}^{-1}$/${\text{I}}_{830\text{cm}}^{-1}$ were related to the exposed or buried status of tryptophan and tyrosine residues, respectively (20). A substantial increase in the ratio of ${\text{I}}_{760\text{cm}}^{-1}$/${\text{I}}_{1003\text{cm}}^{-1}$ representing changes of tryptophan residues was observed during chill storage (*P* < 0.05, Figure 1), indicating transformation of the tryptophan residues from a buried, hydrophobic state to a polar aqueous state (20). The ratio of ${\text{I}}_{850\text{cm}}^{-1}$/${\text{I}}_{830\text{cm}}^{-1}$ significantly increased from 0 to 3 d (*P* < 0.05, Figure 1), indicating an increasing number of exposed tyrosine residues and tryptophan residues. In addition, the ratio of ${\text{I}}_{850\text{cm}}^{-1}$/${\text{I}}_{830\text{cm}}^{-1}$ is also a good indicator for the microenvironment and hydrogen bonds of the ionization of the phenolic hydroxyl group (20). It could be the exposure of polar amino acid residues at the surface of the protein molecules. The ordered and steady structure of the protein was broken into disordered and loose fragments during storage, from a buried status of tryptophan and tyrosine residues to an exposed status, which exposed cleavage sites of digestive enzymes. Thus, we postulate that this disordered and hydrophobic status would increase the degree of protein degradation by exposing more cleavage sites to digestive enzymes.

### The Degree of Protein Degradation in Cooked Meat

**Figure 2:**
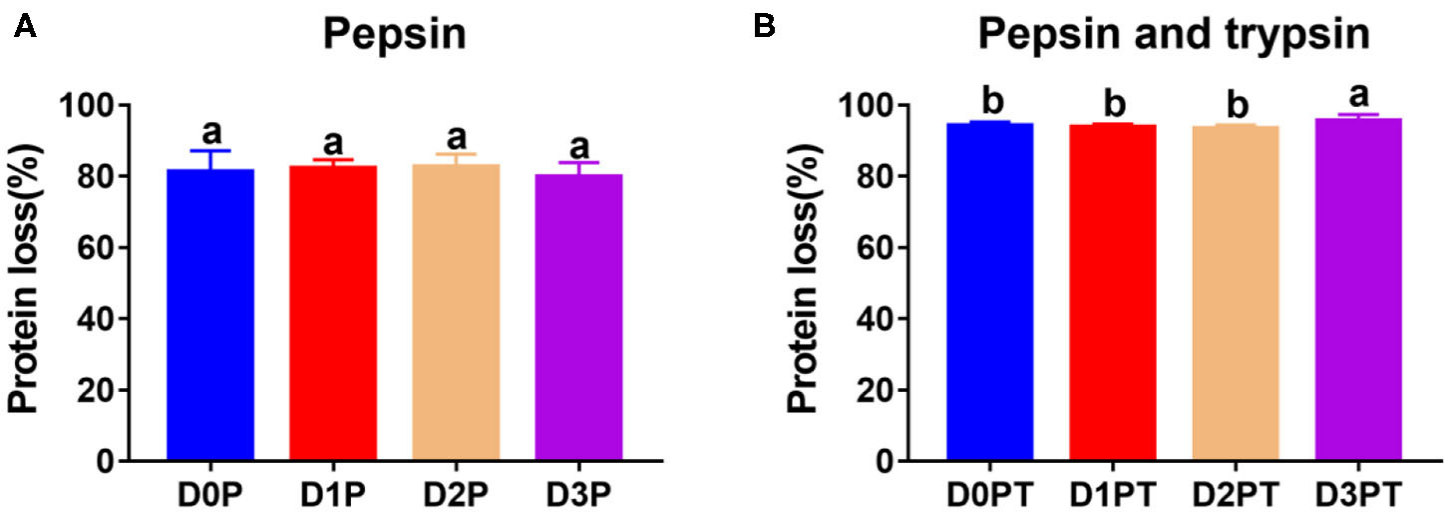
Protein digestibility did not differ after pepsin treatment by differ significantly after pepsin and trypsin treatments. **(A)** D0P, D1P, D2P, and D3P represent 0, 1, 2, and 3 d samples treated by pepsin, respectively. No significant difference was observed among groups (*P* > 0.05). **(B)** D0P/T, D1P/T, D2P/T, and D3P/T represent 0, 1, 2, and 3 d samples treated by pepsin and trypsin, respectively. a, b denotes that protein digestibility differ significantly (*P* < 0.05, *n* = 8 each).

No significant difference was observed in the degree of protein degradation after pepsin digestion among the four time points (*P* > 0.05, Figure 2A). However, the degree of protein degradation increased after two-step digestion, and the 3 d samples had the highest values (*P* < 0.05, Figure 2B). In a previous study, postmortem storage did not improve the *in vitro* digestion parameters of pork (4), in which the structural organization of the muscle cell and the extracellular matrix were not taken into account. To a certain extent, the storage-induced differences in the degree of protein degradation could be attributed to changes in protein secondary or tertiary structures. Such a structural change may further affect protein digestion after meat cooking. Different cooking conditions may affect the structure of meat protein, and the digestibility of meat (17). To avoid it, we cooked meat samples with a similar size for the same time in a water bath. The proteolysis during chill storage may increase protein unfolding and expose more sites to bind to pepsin and trypsin. This is in agreement with the Raman data that reflected structural changes in side chains of proteins, including hydrophobic and electrostatic interactions.

### SDS-PAGE of Proteins in Cooked Meat and Their Digested Products

**Figure 3:**
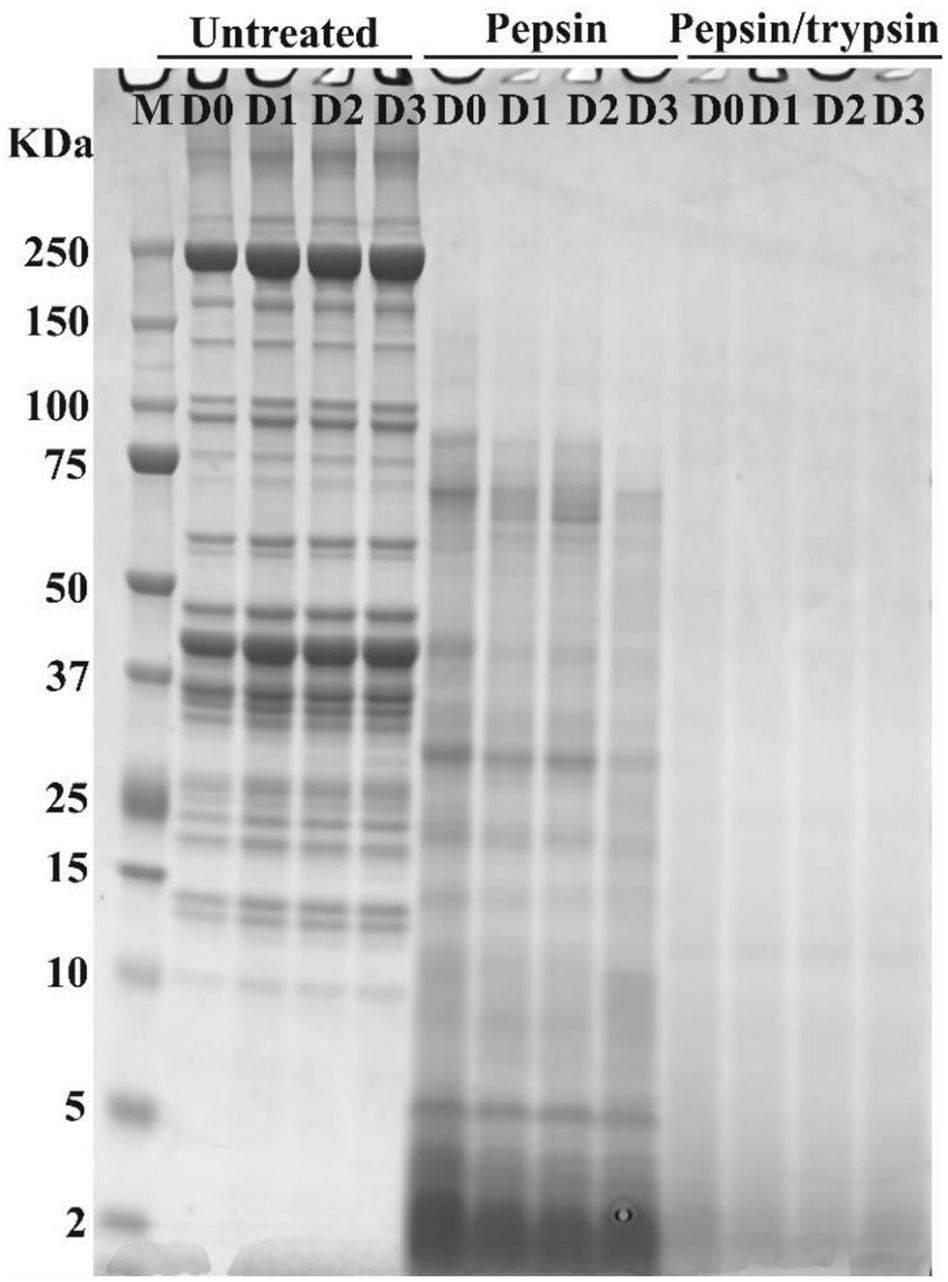
Typical SDS-PAGE patterns of pork proteins before and after digestion. “Untreated” represents total proteins in cooked pork before digestion. Pepsin represents ethanol-insoluble fraction after pepsin digestion. Pepsin/trypsin represents ethanol-insoluble fraction after pepsin and trypsin treatments. Lanes M, D0, D1, D2, and D3 represent calibration marker, 0, 1, 2, and 3 d samples.

SDS-PAGE was applied to separate soluble meat proteins or their digested products before or after pepsin and trypsin treatments (Figure 3). Before digestion (untreated samples), a reduction in band intensity of several protein bands was observed in cooked pork as the storage time of raw meat increased, indicating fragmentation and breakdown of meat proteins. This result was in agreement with our previous study (15). The appearance of the 30 kDa component (Troponin-T fragment) has been considered a good indicator of meat tenderization (21). During postmortem storage, high molecular weight proteins (65–200 kDa) may be degraded into smaller fragments (<25 kDa) (22).

In pepsin treated samples, a substantial decrease in band intensity was observed for some large-molecular-weight protein bands (Figure 3), which could be due to degradation of proteins into smaller peptides or free amino acids. In addition, some insoluble proteins could become soluble after enzymatic digestion (23). Intensity loss or even disappearance of bands was observed for pepsin and trypsin treated samples (Figure 3). Most of large molecular weight bands disappeared because proteins or peptides were further degraded into smaller ones after two-step digestion (Figure 3). Similar phenomena have been observed in different pork cuts and pork products (14, 24). However, SDS-PAGE could not separate smaller peptides well that were characterized by nano LC-MS/MS (15).

### Unique Peptides Could Be a Good Indicator for Chill Storage Time

**Figure 4:**
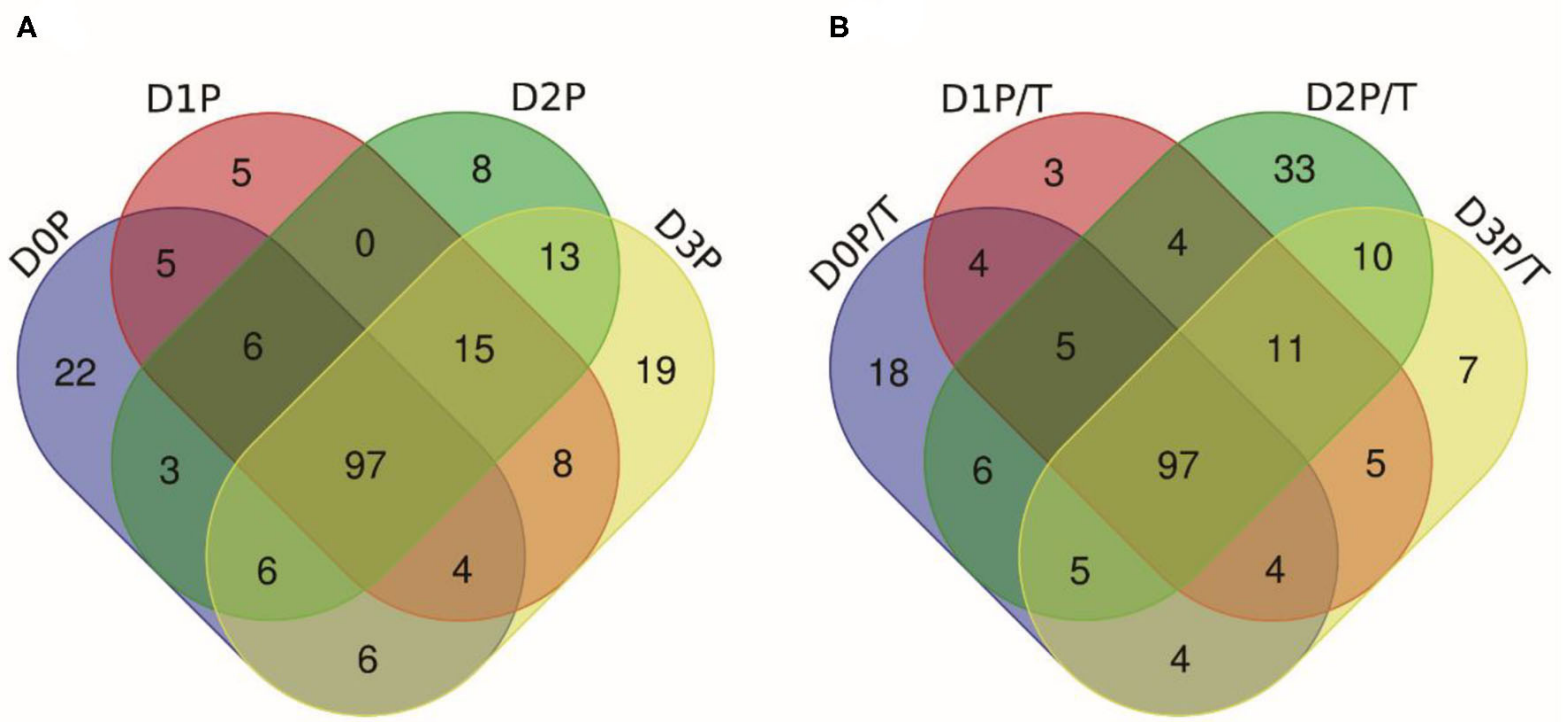
The numbers of unique peptides after pepsin and trypsin treatments. **(A)** D0P, D1P, D2P, and D3P represent 0, 1, 2, and 3 d pepsin treated samples, respectively; **(B)** D0P/T, D1P/T, D2P/T, and D3P/T represent 0, 1, 2, and 3 d pepsin and trypsin treated samples, respectively.

Pepsin or two-step digestion products were identified by nano LC-MS/MS and matched to proteins. All matched proteins are listed in Supplementary Files 1, 2. Venn diagrams revealed that 97 peptides were matched in all pepsin digested samples and pepsin and trypsin digested samples, respectively (Figures 4A,B). Most of these peptides came from myofibrillar proteins (myosin-1, myosin-7, actin) and sarcoplasmic proteins (phosphoglycerate kinase, creatine kinase M-type). In general, peptide abundances decreased as the storage time of raw meat increased, indicating that these peptides were degraded into smaller ones during digestion. Furthermore, 9 peptides matched to myofibrillar proteins, and 52 peptides matched to sarcoplasmic proteins in pepsin-treated samples (Supplementary File 1). More peptides matched to myofibrillar proteins (48/97) than to sarcoplasmic proteins (22/97) (Supplementary File 2). Taken together, the increased protein degradation could be mainly attributed to the degradation of myofibrillar proteins.

Several common peptides appeared in 1, 2, and/or 3 days samples, which were derived from large molecular weight peptides or proteins. Specifically, 22, 5, 8, and 19 peptides were uniquely matched in pepsin-treated samples on 0, 1, 2, and 3 days respectively, and 18, 3, 33, and 6 peptides were unique for pepsin/trypsin-treated samples at the four-time points (Figures 4A,B). Unique peptides could be a good indicator for chill storage time.

### Digested Products From Myofibrillar Proteins in Pork Evolved With Storage Time

All peptides matched to myofibrillar proteins were listed in Table 1. Previous studies have shown that actin, myosin-1, myosin-4, myosin-7, and many glycolytic enzymes are highly abundant proteins in pork muscle (15, 25). These proteins are involved in muscle contraction and energy production. In the present study, most of differently abundant peptides are derived from these proteins (Tables 1, 2).

**Table 1:** Dynamic evolution of peptides derived from myofibrillar proteins after gastric digestion.

**Origin**		**Accession^a^**	**Sequence^b^**	**Modifications**	**MH+ [Da]**	**Area^c^**			
						**D0P**	**D1P**	**D2P**	**D3P**
Myosin	Myosin-1	F1SS64	MNVKHWPWMKL		1469.75				
			MNVKHWPWMKL	M9(Oxidation)	1485.74				
			TVKEDQVFPMNPPKF		1776.90				
			TVKEDQVFPMNPPKF	M10(Oxidation)	1792.90				
			EEAEASLEHEEGKIL		1683.81				
			EEAEASLEHEEGKILRIQL		2194.13				
			ETLKRENKNLQQEISDL		2058.08				
			IRIHFGTTGKL		1242.73				
			EHEEGKILRIQL		1464.82				
			SLIHYAGTVDY		1238.60				
			EDQIISANPLL		1212.65				
			MLTDRENQSIL		1319.66				
			ITGESGAGKTVNTKRVIQYF		2169.16				
			WMVTRINQQL		1288.68				
	Myosin-1 (Fragment)	F1SS62	SELKTKEEEQQRLINDL		2073.08				
			KTKEEEQQRLINDLTAQRARL		2540.39				
	Myosin-7	F1S9D6	MSVKNWPWMKL		1419.73				
			MSVKNWPWMKL	M9(Oxidation)	1435.72				
			ALIHYAGTVDY		1222.61				
			IDSRKGAEKLLGSL		1486.86				
			EEAEASLEHEEGKILRAQL		2152.09				
			RQRYRILNPAAIPEGQF		2029.11				
	MLC2v (Fragment)	A1XQV9	NAFKVFDPEGKGVL		1520.81				
			DYKNLVHIITHGEEKD		1910.96				
			AAFPPDVTGNL		1101.56				
	Myosin light chain	Q29069	RALGTNPTNAEVKKVLGNPSNEEMNAKKIEF		3399.77				
			EAFVKHIMSI	M8(Oxidation)	1190.62				
			EAFVKHIMSI		1174.63				
Troponin	Troponin T fast skeletal muscle type	Q75NG6	YQLEIDKF		1055.54				
Tropomyosin	Tropomyosin TM30-pl (Fragment)	P79309	LEEELKNVTNNL		1415.74				
	Beta-tropomyosin (Fragment)	Q8MKF3	LEEKLKEAETRAEF		1692.88				

a *Protein database ID (Uniprot-Sus scrofa)*.

b *Peptides obtained by MS/MS analysis*.

c *D0P, D1P, D2P, and D3P, 0, 1, 2, and 3 d samples treated by pepsin. Different color indicates different relative abundance of peptides. Red color represents the highest abundance, and the green color represents the lowest abundance*.

**Table 2:** Dynamic evolution of peptides derived from myofibrillar proteins after two-step digestion.

**Origin**	**Accession^a^**	**Sequence^b^**	**Modifications**	**MH+ [Da]**	**Area^c^**				**BIOSEP sequences**	**Activity**
					**D0P/T**	**D1P/T**	**D2P/T**	**D3P/T**		
Myosin										
Myosin-1	F1SS64	EDQVFPMNPPK		1301.62					MNPPK	ACE-inhibitory
		EDQVFPMNPPK	M7(Oxidation)	1317.61					MNPPK	ACE-inhibitory
		LAQESIMDIENEK		1519.73					EK	ACE-inhibitory
		LAQESIMDIENEK	M7(Oxidation)	1535.72					EK	ACE-inhibitory
		GQTVEQVTNAVGALAK		1585.85						
		TVKEDQVFPMNPPKF		1776.90						
		EDQIISANPLL		1212.65						
		IEDEQALALQLQK		1498.81					QK	ACE-inhibitory
		NDLQLQVQAEAEGLADAEER		2199.06						
Myosin-1 (Fragment)	F1SS62	VVESMQSMLDAEIR	M5(Oxidation)	1623.77						
		VVESMQSMLDAEIR		1607.77						
		LAQESTMDIENDKQQLDEK	M7(Oxidation)	2251.04					EK	ACE-inhibitory
		LAQESTMDIENDKQQLDEK		2235.05					EK	ACE-inhibitory
		NLTEEMAGLDETIAK	M6(Oxidation)	1650.79						
		NLTEEMAGLDETIAK		1634.79						
		AGLLGLLEEMR		1201.66						
		AGLLGLLEEMR	M10(Oxidation)	1217.66						
		LQNEVEDLMIDVER	M9(Oxidation)	1718.83						
		LQNEVEDLMIDVER		1702.84						
		LQNEVEDLMLDVER	M9(Oxidation)	1718.83						
		LQNEVEDLMLDVER		1702.83						
		KLETDISQIQGEMEDIIQEAR	M13(Oxidation)	2462.20						
		KLETDISQIQGEMEDIIQEAR		2446.21						
		KKLETDISQIQGEMEDIIQEAR		2574.31						
		NAYEESLDQLETLK		1652.79						
		NAYEESLDQLETLKR		1808.90						
		TKLEQQVDDLEGSLEQEK		2089.03					EK	ACE-inhibitory
		TKLEQQVDDLEGSLEQEKK		2217.13						
		DIDDLELTLAK		1245.66						
		DIDDLELTLAKVEK		1601.86					EK	ACE-inhibitory
		NLQQEISDLTEQIAEGGK		1972.99						
		NLQQEISDLTEQIAEGGKR		2129.08						
		LEQQVDDLEGSLEQEK		1859.89					EK	ACE-inhibitory
		LEQQVDDLEGSLEQEKK		1987.98						
		AITDAAMMAEELK		1393.67						
		AITDAAMMAEELKK		1521.77						
		EFEMSNLQSKIEDEQALAMQLQK		2710.31					QK	ACE-inhibitory
		EFEMSNLQSKIEDEQALAMQLQKK		2838.40						
		AEDEEEINAELTAK		1561.72						
		ALQEAHQQTLDDLQAEEDKVNTLTK		2838.41						
		ANLLQAEIEELR		1398.76						
		IQLELNQVK		1084.63						
		KDIDDLELTLAK		1373.75						
		NDLQLQVQAEADSLADAEER		2215.05						
		SEIQAALEEAEASLEHEEGK		2170.02						
		SQEDLKEQLAMVER		1675.83						
		VAEQELLDASER		1359.68						
		SLIHYAGTVDY		1238.60						
		DTQLHLDDALR		1296.66						
		DTQIHLDDALR		1296.65						
		IEELEEEIEAER		1488.71						
		IEELEEELEAER		1488.71						
		MEGDLNEMEIQLNHANR		2013.91						
		RANLLQAEIEELR		1554.86						
		TLEDQLSELK		1175.62						
		MNVKHWPWMKL		1469.76						
		IEKPMGIF		934.51						
		NTQGILKDTQIHLDDALR		2051.08						
		TNEKLQQFF		1154.58						
		ENKNLQQEISDLTEQIAEGGKR		2500.27						
		IEDEQALAMQLQK		1516.77					QK	ACE-inhibitory
		KAITDAAMMAEELKK	M9(Oxidation)	1665.85						
		KALQEAHQQTLDDLQAEEDKVNTLTK		2966.51						
		KMEGDLNEMEIQLNHANR		2142.01						
Myosin-7	F1S9D6	NLTEEMAGLDEIIAK		1646.83						
		NLTEEMAGLDEIIAK	M6(Oxidation)	1662.83						
		LEDEEEMNAELTAK		1621.72						
		LEDEEEMNAELTAK	M7(Oxidation)	1637.72						
		VKLEQHVDDLEGSLEQEK		2096.06					EK	ACE-inhibitory
		VKLEQHVDDLEGSLEQEKK		2224.15						
		LEQHVDDLEGSLEQEK		1868.89					EK	ACE-inhibitory
		LEQHVDDLEGSLEQEKK		1996.98						
		ALQEAHQQALDDLQAEEDKVNTLTK		2808.40						
		DFELNALNAR		1162.59						
		LAEQELIETSER		1417.72						
		LTQESIMDLENDKQQLDER		2305.10						
		NDLQLQVQAEQDNLADAEER		2299.08						
		NLQEEISDLTEQLGSSGK		1947.95						
		NNLLQAELEELR		1441.77						
		LQDLVDKLQLK		1312.78						
		DTQIQLDDAVR		1273.64						
		LQNEIEDLMVDVER		1702.83						
		LELQSALEEAEASLEHEEGK		2212.06						
Myosin light chain 1/3	A1XQT6	KPAAAAAPAPAPAPAPAPAPAPPK		2098.18					PK	DPPIV inhibitory
		KPAAAAAPAPAPAPAPAPAPAPPKEEK		2484.36					EK	ACE-inhibitory
		ITLSQVGDVLR		1200.71						
		DQGSYEDFVEGLR		1514.68						
Myosin heavy chain	Q95249	KLETDISQIQGEMEDIVQEAR		2432.20						
		KLETDISQIQGEMEDIVQEAR	M13(Oxidation)	2448.19						
Myosin, heavy chain 7	H6SHX6	LGSLDIDHNQY		1274.60						
Actin										
Actin (Fragment)	B2ZFN7	IGMESAGIHETTY	M3(Oxidation)	1424.64						
		IGMESAGIHETTY		1408.64						
		MKILTERGYSF		1344.70						
		MKILTERGYSF	M1(Oxidation)	1360.69						
		FQPSFIGMESAGIHETTY		2014.92						
		QPSFIGMESAGIHETTY		1867.85						
		SYELPDGQVITIGNER		1790.89						
Tropomyosin										
Tropomyosin 4	D0G7F7	MEIQEMQLK	M6(Oxidation)	1165.56						
		MELQEMQLK		1149.56						
		LVILEGELER		1170.67						
		KLVILEGELER		1298.77						
Tropomyosin alpha-1 chain	F2Z5B6	AISEELDHALNDMTSI	M13(Oxidation)	1774.82						
		AISEELDHALNDMTSI		1758.82						
		MELQEIQLK	M1(Oxidation)	1147.60						
		MELQEIQLK		1131.61						
		KLVIIESDLER		1314.76						
		LVIIESDLER		1186.67						
		IQLVEEELDR		1243.65						
		IQLVEEELDRAQER		1727.89						
		QLEDELVSLQK		1301.70					QK	ACE-inhibitory
		SKQLEDELVSLQK		1516.82					QK	ACE-inhibitory
		KAISEELDHALNDMTSI		1886.91						
Tropomyosin 3	Q2XQY5	LVIIEGDLER		1156.66						
Troponin										
Troponin I	B3VI70	SVMLQIAATELEK		1432.77					EK	ACE-inhibitory
troponin C	A1XQV5	AAFDMFDADGGGDISVK		1715.76						
		SYLSEEMIAEFK		1446.68						
Troponin T	Q75NG6	YQLEIDKF		1055.54						

a *Protein database ID (Uniprot-Sus scrofa)*.

b *Peptides obtained by MS/MS analysis*.

c *D0PT, D1PT, D2PT, and D3PT, 0, 1, 2, and 3 d samples treated by pepsin and trypsin. Different color indicates different relative abundance of peptides. Red color represents the highest abundance, and the green color represents the lowest abundance*.

Oxidative modification happened in four peptides (MNVKHWPWMKL, EAFVKHIMSI TVKEDQVFPMNPPKF, MSVKNWPWMKL) originating from myosin. Long-chain peptides (e.g., EEAEASLEHEEGKILRIQL) were less abundant than short-chain peptides (e.g., EEAEASLEHEEGKIL) and some long-chain peptides disappeared in 2nd days samples (Table 1). After gastric digestion, unique peptides were further hydrolyzed by trypsin. Abundances of common peptides (ALIHYAGTVDY, TVKEDQVFPMNPPKF, LEQQVDDLEGSLEQEK, VKLEQHVDDLEGSLEQEK, KPAAAAAPAPAPAPAPAPAPAPPK, EDQVFPMNPPK) decreased as the storage time of raw meat increased, indicating that these peptides were digested by pepsin or/ and trypsin (Tables 1, 2). A total of 16 peptides, including KPAAAAAPAPAPAPAPAPAPAPPK, KPAAAAAPAPAPAPAPAPAPAPPKEEK, TKLEQQVDDLEGSLEQEK, and TKLEQQVDDLEGSLEQEKK were cleaved into 1 to 4 amino acids peptides by trypsin (Table 2).

From the digested products, 117 peptides were selected to match the BIOPEP database showing ACE-inhibitory (angiotensin-converting-enzyme inhibitory) and dipeptidyl peptidase IV (DPPIV) inhibitory activities (Table 2). Eighteen peptides were estimated to have ACE-inhibitory and DPPIV inhibitory activities, which have been identified in the database (26). ACE inhibitors are known to beneficial to treat arterial hypertension (27), diabetes mellitus, ischemic heart disease, chronic heart failure, angioedema, chronic kidney disease, and Parkinson's disease (28, 29). DPPIV inhibitor may be beneficial for NASH subjects (30). Four peptides (TKLEQQVDDLEGSLEQEKK, LEQQVDDLEGSLEQEKK, VKLEQHVDDLEGSLEQEKK, LEQHVDDLEGSLEQEKK) were identified, which contain sequences VDDLEGSLEQEKK and DDLEGSLEQEKK that were previously reported to have antioxidant peptides (31). It is of note that some identified peptides maintain a long sequence after the two-step digestion. However, almost all the peptides would be hydrolyzed by peptidases into smaller fragments (dipeptides and tripeptides) locating the outer layer of intestinal epithelium before reaching the blood stream (32). Thus, moderate chill storage may enhance the production of antioxidant, ACE inhibitor and DPP-IV inhibitory peptides.

Table 2 illustrates dynamic evolution of 117 peptides derived from cooked meat samples. The potential bioactive peptides were predominantly originated from myosin, actin, tropomyosin and troponin. Peptides EDQVFPMNPPK (Oxidation) and IEDEQALALQLQK appeared in 0 day samples, whereas peptides EFEMSNLQSKIEDEQALAMQLQK, IEDEQALAMQLQK, and SKQLEDELVSLQK appeared only in 2 day samples. Abundances of LAQESIMDIENEK, LAQESTMDIENDKQQLDEK, and their oxidative peptides and KPAAAAAPAPAPAPAPAPAPAPPKEEK were the lowest in 2 day samples. Abundances of SVMLQIAATELEK, DIDDLELTLAKVEK, and TKLEQQVDDLEGSLEQEK were the highest in 2 day samples. This could be mainly attributed to the degradation of proteins and accumulation of peptides in the first stage, which would be further degraded into smaller peptides that have ACE and DPPIV inhibitory bioactivities. Thus, 2 days could be a key time point for chill storage of pork in terms of meat nutrition.

Two peptides were observed to have the same sequence but their molecular weights were different, which is attributed to protein oxidation based on the database. Eighteen peptides from two-step digestion were observed to have oxidation modification. It is known that protein oxidation occurs during storage and the extent of protein oxidation may be enhanced as meat storage time increases. In the present study, pork samples were vacuum packed and stored at low temperature (4°C) for 3 days. In such a condition, protein oxidation could be alleviated. And thus more cleavage sites could be exposed in loose protein structure and protein degradation increased. The number of bioactive peptides may increase after the two-step digestion.

The above results could be associated with rigor mortis and subsequent aging of muscle (33). It is well-known that glycolysis occurs in skeletal muscles after slaughter (34, 35). This process is accompanied by the dephosphorylation of energy metabolic enzymes, the formation of actomyosin, and the shortening of sarcomeres (6, 36). Such activities may make some energy metabolic enzymes and myofibrillar proteins less susceptible to pepsin and trypsin digestion under *in vitro* condition on day 1. However, prolonged storage can tenderize meat, and make the myofibrillar proteins and bound energy metabolic enzymes more digestible. This is because the endogenous enzymes, e.g., μ-calpain and cathepsins, catalyze the degradation of myofibrillar proteins into smaller fragments (33), which will expose the cleavage sites for digestive enzymes. It is notable that, too long-time storage of fresh meat may have the problems of food spoilage, discoloration, and the decreased digestibility of meat proteins due to oxidation (37).

## Conclusions

In this study, postmortem aging was shown to have significant impacts on the structural characteristics and *in vitro* digestion of pork proteins. It is observed that the 3 d samples had lower α-helix content and the peak intensity at 760 cm^−1^ (tryptophan residues), but higher β-sheet, β-turn, and random coil contents than the 0 d samples. This is accompanied by significant protein degradation and increased susceptibility to digestion. The structural changes of proteins altered the accessibility of proteolytic enzymes to the cleavage sites, and consequently the protein digestion. Further work is necessary to evaluate the effect of postmortem aging on *in vivo* digestion of pork protein.

## Data Availability Statement

The datasets generated for this study can be found in Proteome Xchange, Accession No. PXD020595.

## Ethics Statement

The animal study was reviewed and approved by the Ethical Committee of Experimental Animal Center of Nanjing Agricultural University.

## Author Contributions

CL designed the experiments. XZ, JH, DZ, MZ, YX, and CW conducted the experiments. XZ, JH, and CL wrote the paper. All authors have read and approved the manuscript.

## Conflict of Interest

The authors declare that the research was conducted in the absence of any commercial or financial relationships that could be construed as a potential conflict of interest.
